# Lack of Pattern Separation in Sensory Inputs to the Olfactory Bulb during Perceptual Learning

**DOI:** 10.1523/ENEURO.0287-17.2017

**Published:** 2017-09-27

**Authors:** Monica W. Chu, Wankun L. Li, Takaki Komiyama

**Affiliations:** Neurobiology Section, Center for Neural Circuits and Behavior, and Department of Neurosciences, University of California, San Diego, La Jolla, CA 92093

**Keywords:** mitral cells, olfactory bulb, olfactory sensory neurons, perceptual learning, plasticity, two-photon imaging

## Abstract

Recent studies revealed changes in odor representations in the olfactory bulb during active olfactory learning (Chu et al., 2016; Yamada et al., 2017). Specifically, mitral cell ensemble responses to very similar odorant mixtures sparsened and became more distinguishable as mice learned to discriminate the odorants over days (Chu et al., 2016). In this study, we explored whether changes in the sensory inputs to the bulb underlie the observed changes in mitral cell responses. Using two-photon calcium imaging to monitor the odor responses of the olfactory sensory neuron (OSN) axon terminals in the glomeruli of the olfactory bulb during a discrimination task, we found that OSN inputs to the bulb are stable during discrimination learning. During one week of training to discriminate between very similar odorant mixtures in a Go/No-go task, OSN responses did not show significant sparsening, and the responses to the trained similar odorants did not diverge throughout training. These results suggest that the adaptive changes of mitral cell responses during perceptual learning are ensured by mechanisms downstream of OSN input, possibly in local circuits within olfactory bulb.

## Significance Statement

Odor representations in the rodent olfactory bulb have been demonstrated to undergo dynamic changes during a variety of olfactory experiences. In particular, odor representations in mitral cells, the primary projection neurons of the olfactory bulb, become more different (pattern separation) when mice are trained to distinguish between two similar odor mixtures. Here, we address whether similar changes occur in olfactory sensory neurons (OSNs), which provide input onto mitral cells. We find that OSN odor representations do not exhibit pattern separation when mice are trained to discriminate between two similar odor mixtures, suggesting that the changes likely occur downstream of OSNs during this task.

## Introduction

Olfactory transduction occurs when odorants bind to specific receptors expressed by olfactory sensory neurons (OSNs), each of which expresses only one of ∼ 1000 types of odorant receptors in the nasal epithelium (Ressler et al., 1994; Vassar et al., 1994; Mombaerts et al., 1996). Axons from OSNs expressing the same type of receptor converge onto one or two glomeruli on the surface of the olfactory bulb; thus different odorants are represented at this early stage in olfactory encoding by unique combinations of activated glomeruli. At the glomerular layer, OSNs provide input onto mitral/tufted cells, the primary projection neurons of the olfactory bulb, which in turn send their axons to higher brain areas (Price and Powell, 1970). Additionally, throughout the olfactory bulb, inhibitory interneurons play a major role in transforming odor representations (Lledo et al., 2008; Mcgann, 2013; Imai, 2014; Yu et al., 2014).

Odor representations in the early olfactory system are highly dynamic and have been shown to be modulated by various types of olfactory experience. For example, the activity of OSNs can change with passive experience (Kass et al., 2016) or associative learning, such as fear conditioning (Kass et al., 2013) or odor-reward association (Abraham et al., 2014). Experience has also been shown to induce response changes further downstream in the olfactory circuit: changes in odor responses in inhibitory interneurons of the olfactory bulb, which are targets of feedback from cortical and neuromodulatory areas (Ma and Luo, 2012; Nunez-Parra et al., 2013; Boyd et al., 2015; Otazu et al., 2015), have been shown to correlate with odor-specific changes in behavior (Mandairon et al., 2008; Moreno et al., 2009). Additionally, mitral cell activity can also be modulated by passive experience or behavioral training (Kay and Laurent, 1999; Doucette and Restrepo, 2008; Doucette et al., 2011; Kato et al., 2012; Li et al., 2015; Yamada et al., 2017).

In particular, we recently showed that mitral cell odor ensembles undergo bidirectional changes in odor representations during week-long discrimination learning. Specifically, when mice learned to discriminate between very different odorants, the representations of the odorants became more similar to each other. In contrast, discrimination learning of very similar odorants led to an enhanced separation of the odor representations, correlating with the improved ability of mice to discriminate the similar odorants (Chu et al., 2016). These mitral cell response changes could arise from at least two different mechanisms. First, mitral cells may simply reflect the changes that occur at the level of OSN inputs. Second, plasticity downstream of OSN inputs may alter the way mitral cells respond to the same OSN inputs. To begin to distinguish these possibilities, here, we addressed whether OSN inputs show plasticity during the same paradigm in which mice learn to discriminate between very similar odorant mixtures. This was achieved by longitudinal two-photon calcium imaging to characterize the activity of OSN axon terminals in behaving mice. We find that although mice exhibited a behavioral improvement in their ability to discriminate between odorants during training, OSN odor representations remained relatively stable throughout training without exhibiting enhanced pattern separation, suggesting that learning-related plasticity likely occurs downstream of OSNs.

## Materials and Methods

### Animals

All procedures were in accordance with protocols approved by the UCSD Institutional Animal care and Use Committee and guidelines of the National Institutes of Health. Transgenic mice were obtained from Jackson laboratories [OMP-tTA, RRID:IMSR_JAX:017754 (Yu et al., 2004) and tetO-GCaMP6s, RRID:IMSR_JAX:024742 (Wekselblatt et al., 2016)] and group housed in disposable plastic cages with corncob bedding in a reversed light cycle room (12/12 h). Experiments were all performed during the dark period.

### Surgeries

Surgeries were performed in adult male mice (at least six weeks old) as described previously (Kato et al., 2012). Briefly, mice were anesthetized with isoflurane, the skull was exposed and a steel headplate was glued to the skull. The skull above the olfactory bulb was then removed and replaced with an optical glass window (∼1 × ∼2 mm), which was secured in place with dental cement. Mice were then allowed to recover for at least two weeks (35 ± 9.3 d, mean ± SEM) before imaging.

### Odorant delivery

Odorants (Sigma) were delivered to the mouse through a Teflon-coated tube at a final concentration of 100 ppm and flow rate of 1 l/min. Odorant vials contained odorant diluted in mineral oil (Thermo Fisher) to a calculated vapor pressure of 200 ppm, and a custom olfactometer mixed odorant vapor from odorant vials with filtered and humidified house air at 1:1 to achieve a final concentration of 100 ppm.

### Behavioral training

The training protocol, including the duration, criteria and odorant choice, was identical to our previous study (Chu et al., 2016). Mice began water restriction (∼1 ml/d) ∼3-5 d after surgery, and weight was maintained at 80-85% of initial weight. Mice first went through a pretraining period, where they became accustomed to being head-fixed in the imaging environment and learned the Go/No-Go olfactory discrimination task.

The behavioral task was controlled by a real-time program (C. Brody). In the olfactory discrimination task, mice performed daily sessions that lasted 150 trials, or until the mouse disengaged, whichever came first. Mice performed 140.0 ± 2.13 trials/d (mean ± SEM). In each trial, one of two odorants was pseudorandomly delivered (maximum of three trials in a row with the same odorant). Each trial consisted of a 4-s odorant delivery period, followed by a 2-s answer period, during which the mouse could choose whether or not to lick a lickport. A water reward (∼6 μl water) was delivered from the lickport if the rewarded odorant (S+) was delivered in a trial and the mouse responded by licking the lickport during the answer period. Any other action, such as not licking to S+ or the unrewarded odorant (S-), or licking to S-, did not result in a water reward. A 15-s intertrial interval followed the answer period, and there was no punishment delivered for error trials (not licking to S+, or licking to S-).

In the first phase of training, called pretraining (∼7-14 d), mice were trained with citral (S+) and limonene (S-). The intertrial interval (ITI) started at 3 s and as mice performed above 80% success rate, the ITI was increased incrementally by 2 s every half-session until the ITI reached 15 s. Once the mice performed above 80% success rate for the first odorant pair (citral/limonene), they were trained with a second pair, +-carvone (S+) and cumene (S-) until they reached above an 80% success rate.

Pretraining was followed by the week-long difficult discrimination task, during which glomerular activity was imaged while mice were performing the same Go/No-Go task with a novel set of similar binary odorant mixtures consisting of heptanal (Sigma, W254002) and ethyl tiglate (Sigma, W246000). S+ was a mixture of 52% heptanal 48% ethyl tiglate, while S- was a mixture of 48% heptanal 52% ethyl tiglate.

### Imaging

Two-photon imaging was performed with a commercial microscope (B-scope, Thorlabs) with 925 nm excitation from a Ti-Sa laser (Spectra-physics). Images were acquired at a framerate of ∼28 Hz. Each imaging frame consisted of 512 × 512 pixels, and spanned 1132 × 982 μm. Images were acquired for ∼2.4-min-long segments, with intersegment intervals of 7 s. Trials with missing data (trials which overlapped with the intersegment intervals) were not analyzed. A custom (MATLAB, RRID:SCR_001622) program performed motion correction (Full-frame cross-correlation correction) on imaging frames.

#### Data analysis

In two sessions, we were not able to collect imaging data, and these sessions were excluded from analysis (day 3: one mouse; day 6: one mouse).

#### Regions of interest (ROIs)

ROIs were manually drawn around individual glomeruli, using a reference image which was created from a maximum projection of the imaged data on the first day of the experiment. For subsequent days, ROIs were shifted manually to accommodate shifts in alignment from day to day. All fluorescence pixel values within each ROI were averaged to create a fluorescence time series. For each trial, the time series for a single glomerulus was normalized to the baseline period for that trial (the 5 s preceding odor onset) to calculate the dF/F. The total number of animals imaged was 13 mice, and 32 ± 2.38 (mean ± SEM) glomeruli were imaged in each mouse.

#### Classifying glomeruli as divergent

A glomerulus was divergent if the following criteria were met. Criterion 1: dF/F is significantly different (*p* < 0.05) between odorant trials for >75% of time points within a sliding half-second window during the odorant period. A Wilcoxon rank sum test was performed to compare odorant 1 and odorant 2 dF/F values for each time frame. Criterion 2: the difference between trial-averaged dF/F trace for odorant 1 and odorant 2 exceed 0.2 in at least one time point during the sliding half-second window which meets the first criterion.

#### Classifying glomeruli as responsive

A glomerulus was classified as responsive if it was classified as divergent, and/or if the following criteria were met. Criterion 1: dF/F is significantly different (*p* < 0.05) for >75% of time points within a sliding half-second window during the odorant period. A Wilcoxon rank sum test was performed to compare all baseline values (0–5 s of all trials) dF/F values with each time frame during the odor response period. Criterion 2: the difference between trial-averaged dF/F trace for odorant 1 and odorant 2 exceed 0.2 in at least one time point during the sliding half-second window which meets the first criterion.

#### Calculating d-prime

All trial traces were smoothed with the MATLAB “smooth” function with a time constant of six frames (∼0.25 s). The sensitivity index d-prime was calculated for divergent glomeruli: d’ = |*mean dF/F_odorant 1_* − *mean dF/F_odorant 2_*| / *pooled standard deviation_odorant 1 and 2_*.

If a glomerulus was divergent on a given day, d’ was calculated for each time frame during the odorant period (0–4 s after odorant onset). The maximum d’ value of all frames during the odorant period was assigned to each glomerulus, and the average of these maximum d’ values of divergent neurons for each mouse on each session was calculated and plotted in Figure 3*A*.

##### Glomerular activity vectors

For each mouse, decoder analysis was performed using 100 iterations. For each iteration, 14 glomeruli were randomly selected from all glomeruli that were responsive to at least one odorant in at least 1 d. The subset size of *n* = 14 glomeruli was determined by the mouse with the lowest number of responsive glomeruli.

For each trial, an activity vector was assembled by concatenating the average dF/F from 0–2 s and 2–4 s bins during the odor period. A single mouse would then have a 28-dimensional activity vector (14 glomeruli × two time bins per glomeruli).

##### Nearest-centroid decoder

The decoder accuracy was calculated as follows. Each trial was classified by assigning that trial with the identity of the odor with the closest centroid in glomerular activity space. The trial that was being classified was excluded from the odor centroid calculation. The decoder accuracy for each iteration for each day is then calculated as fraction of trials that were successfully classified by the decoder. The final decoder accuracy value is calculated as the mean of the 100 iterations.

##### Correlation coefficient

For each day and odor, a mean odor activity vector was created by averaging across trials for each odorant. The correlation coefficient between the means of the population activity vectors was calculated using the Matlab function “corrcoef.” The mean correlation coefficient value across animals is plotted in Figure 3*B*.

##### Peak amplitude of odorant response

For glomeruli that were classified as having excitatory odorant responses, the peak amplitude was calculated as the maximum dF/F between a time window of 0–8 s after odorant onset (Fig. 3*C*,*D*).

## Results

### Chronic imaging of OSN activity in behaving mice

**Figure 1.**
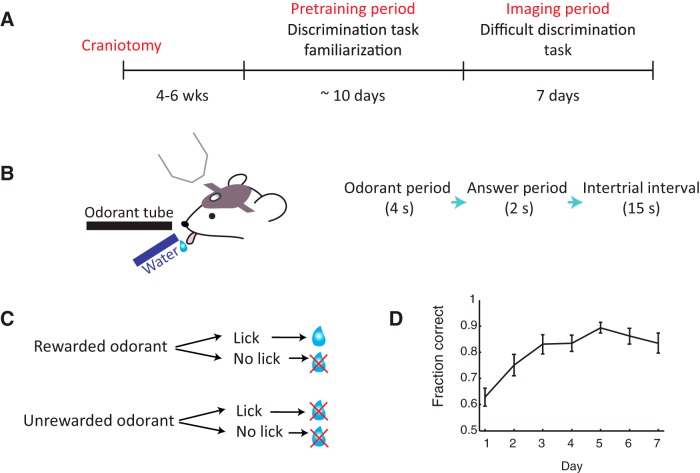
Training mice to perform a difficult discrimination task. ***A***, Experimental timeline. Water-restricted mice first undergo a pretraining period, where they become familiarized with the discrimination task using easy odorant pairs. After the pretraining period, glomerular responses are imaged while the mouse learns to perform a difficult discrimination task with two similar odor mixtures. ***B***, Schematic of imaging setup (left) and trial structure (right). ***C***, Schematic of behavioral paradigm. If a rewarded odorant is presented and the mouse responds with a lick, a water reward will be delivered through the lickport. If the unrewarded odor is delivered, no water reward will be given regardless of the mouse’s actions. ***D***, Fraction of correctly answered trials (mean ± SEM of all mice, *n* = 13 mice) on each day of difficult discrimination training. Mice take 3 d on average to perform above an 80% success rate.

We recently showed that when mice are trained to discriminate between very similar odorants, mitral cell odor representations for the similar odorants become more separable (Chu et al., 2016). In the current study, we addressed whether OSN inputs to the olfactory bulb exhibit plasticity during the same task. More specifically, we asked if training mice to discriminate between two similar odorants would enhance the separation of OSN responses to the two odorants. To record OSN activity, we used two-photon imaging of OSN axon terminals at the glomerular layer of the olfactory bulb in transgenic mice expressing GCaMP6s specifically in mature OSNs (OMP-tTA::tetO-GCaMP6s). Craniotomies were performed above the right olfactory bulb, and after a recovery period, mice were trained to learn a Go/No-Go discrimination task (Fig. 1*A*). First, mice underwent a pretraining period, where they learned the Go/No-Go paradigm in which mice were presented in each trial with one of two very different odorants, only one (S+) of which was paired with a water reward. Mice eventually learned to lick the lickport in response to S+ to obtain a water reward, while withholding from licking to the unrewarded odorant (S-; Fig. 1*B*,*C*). After reaching a success rate of above 80% correct in the Go/No-Go task with the pretraining odorants, mice were then trained to perform a difficult discrimination task in which they were required to discriminate between two highly similar binary odorant mixtures (odorant 1: S+, 52% heptanal and 48% ethyl tiglate; odorant 2: S-, 48% heptanal and 52% ethyl tiglate; % are of total concentration of 100 ppm).

Throughout the week-long training in the difficult discrimination task, mice gradually improved their performance, taking on average ∼3 d (3.18 ± 0.51; mean ± SEM) to reach a level of expertise above an 80% success rate (Fig. 1*D*). The speed of learning was comparable to the same discrimination task in Chu et al., 2016 (Wilcoxon rank sum test, *p* = 0.15) and require more days to reach expertise compared to the discrimination of very distinct odor pairs [also from Chu et al. (2016), Wilcoxon rank sum test, *p* = 0.0008], indicating the requirement of perceptual learning in the difficult discrimination task.

### Glomerular odor responses do not sparsen during training

**Figure 2.**
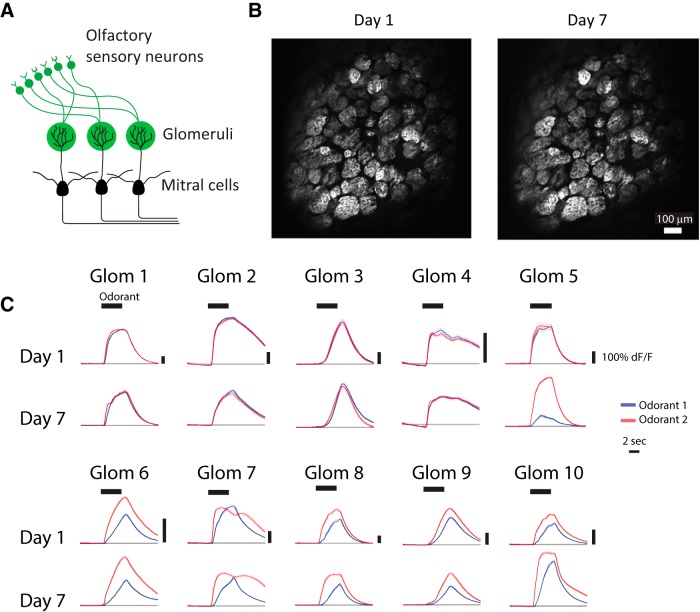
Imaging glomerular odor responses during training. ***A***, Schematic of the olfactory bulb. Two photon imaging of glomerular responses was performed in OMP-tTA::tetO-GCaMP6s mice, in which OSNs express GCaMP6s. ***B***, An example of a typical glomerular field of view on the first day of imaging (day 1) and 6 d later (day 7). ***C***, Examples of odorant responses (mean ± SEM) from individual glomeruli. Responses to the odorant 1 (S+, rewarded odorant) are shown in blue, and responses to odorant 2 (S-, unrewarded odorant) are shown in black. Odorant period is indicated by the thick horizontal black bar.

**Figure 3.**
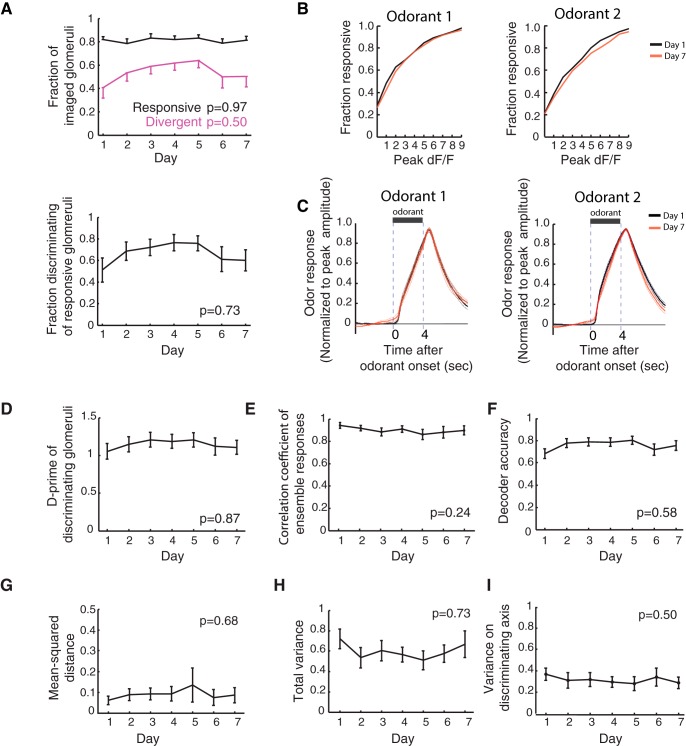
Glomerular odor representations do not show an increased separation during training. ***A***, top, Fractions of glomeruli classified as responsive (black) and divergent (magenta) are plotted for each day. The responsive fraction and divergent fraction remain stable across days (Pearson correlation; responsive, *p* = 0.97; divergent, *p* = 0.50). Bottom, Fraction of divergent glomeruli out of all responsive glomeruli does not significantly change during training (Pearson correlation; *p* = 0.73). ***B***, Cumulative fraction distributions of the peak amplitude of excitatory glomerular odorant responses on day 1 and day 7. There is no difference in the distributions for either odorant between day 1 and day 7 (Kolmogorov-Smirnov test: odorant 1, *p* = 0.64; odorant 2, *p* = 0.99). ***C***, Mean excitatory glomerular odorant responses on day 1 (black) and day 7 (red), normalized to peak amplitude. There are no significant differences between responses on day 1 and day 7 (Wilcoxon rank sum test for each imaging frame, false-discovery rate corrected: *q* = 0.05). ***D***, The averaged d-prime of all divergent glomeruli does not change with difficult discrimination training (Pearson correlation; *p* = 0.87). ***E***, The correlation coefficient between averaged ensemble odorant responses does not change with difficult discrimination training (Pearson correlation; *p* = 0.24). ***F***, The decoder accuracy does not change during difficult discrimination training (Pearson correlation; *p* = 0.58). ***G–I***, There were no significant changes in the (***G***) mean-squared distance between odor centroids nor (***H***) the total variance across trials for each odor (Pearson correlation; mean-squared distance, *p* = 0.68; total variance, *p* = 0.73). ***I***, There was also no change in the variance along the axis of discrimination (the axis containing the line connecting Odor 1 and Odor 2 centroids) for the decoder (Pearson correlation; *p* = 0.50). Unless otherwise stated, mean ± SEM are shown in line plots with error bars.

To characterize OSN activity in behaving mice, we used two-photon calcium imaging to monitor the activity of OSN axon terminals in glomeruli throughout the learning of the difficult discrimination task (Fig. 2*A*,*B*). We tracked the activity in 32 ± 2.4 (mean ± SEM) glomeruli in each mouse. Individual glomeruli exhibited odor responses by fluorescence changes during the 4-s odor period, with predominantly excitatory responses (Fig. 2*C*). With some exceptions, individual glomeruli showed stable odorant responses during difficult discrimination training (Fig. 2*C*). As a population, the fraction of responsive glomeruli was stable throughout training (Pearson correlation, *p* = 0.97; Fig. 3*A*), and there was no change in the response amplitude of responsive glomeruli across days as a population (Kolmogorov-Smirnov test: odorant 1, *p* = 0.64; odorant 2. *p* = 0.99; Fig. 3*B*). Additionally, the temporal dynamics of the glomerular odor responses remained stable for each odorant from day 1 to day 7 (Wilcoxon rank sum test for each imaging frame, false-discovery rate corrected: *q* = 0.05, N.S.; Fig. 3*C*). This is reminiscent of the stable level of OSN odor responses observed during a week-long passive exposure by imaging synaptopHlourin responses in OSN terminals (Kato et al., 2012) and in contrast to the profound sparsening observed in mitral cell responses over a week-long passive exposure (Kato et al., 2012) and discrimination learning (Chu et al., 2016). These results suggest that the sparsening of mitral cell responses during passive exposure and discrimination learning is likely due to changes downstream of OSN input onto mitral cells.

### No increase in separation of OSN odor responses during discrimination learning

In addition to the sparsening, mitral cell responses to the similar odorants showed an enhanced separation during the discrimination task (Chu et al., 2016). We then asked whether OSN responses showed similar changes, potentially underlying the mitral cell changes and the behavioral improvement during learning. However, in contrast to what has been observed with mitral cell ensembles (Chu et al., 2016), there was no significant change in the fraction of glomeruli whose activity distinguished the two odorants (“divergent glomeruli”) across days during training (Pearson correlation; divergent, *p* = 0.50; divergent of responsive, *p* = 0.73; Fig. 3*A*). Furthermore, in divergent glomeruli, the degree of divergence (quantified as d-prime) remained stable throughout training (Pearson correlation; *p* = 0.87; Fig. 3*D*). Thus, we found no evidence for an enhanced separation of responses to the two similar odorants at the level of individual glomeruli.

Next, we investigated potential changes at the level of glomerular ensembles. To address this, we first calculated the correlation coefficient as a measure of similarity between glomerular ensemble response vectors. Consistent with what was observed in individual glomeruli, the correlation coefficient between glomerular odor responses was stable throughout training (Pearson correlation; *p* = 0.24; Fig. 3*E*). We next performed a linear decoder analysis to assess the discriminability between glomerular odor representations. The average decoder accuracy, or the fraction of correctly decoded trials on each day, remained stable during training (Pearson correlation; *p* = 0.58; Fig. 3*F*). Consistent with the stable correlation coefficient and decoder accuracy, we found no change in the distance between the mean responses for the two odorants in glomerular activity space (Pearson correlation; *p* = 0.68; Fig. 3*G*). We also did not observe any change in the total variance across same odor trials in glomerular activity space (Pearson correlation; *p* = 0.73; Fig. 3*H*), nor in the variance along the decoder axis of discrimination (Pearson correlation; *p* = 0.50; Fig. 3*I*). Thus, although mice exhibited a behavioral improvement in their ability to discriminate the two odorants during training, multiple measures of discriminability by glomerular ensembles indicate that OSN odor responses did not show an enhanced separation throughout training. Therefore, it seems unlikely that OSNs are a main source of the plasticity observed in mitral cell ensembles (Chu et al., 2016) during the learning of this difficult discrimination task.

## Discussion

Recent studies have revealed changes in mitral cell responses during discrimination learning paradigms. In particular, the learning to discriminate between very similar odorants led to an enhanced pattern separation in mitral cell responses, potentially underlying the perceptual learning (Abraham et al., 2014; Chu et al., 2016). Here, we explored whether the mitral cell changes may be inherited from changes in OSN inputs to the bulb. However, the separation of odor responses of OSN inputs remained stable during the same week-long learning paradigm that induced an enhanced separation of mitral cell responses, suggesting that plasticity during this task occurs downstream of OSN input onto mitral cells.

We acknowledge that several previous studies have demonstrated changes in the glomerular activity during olfactory experiences. For example, week-long passive exposure to a single odorant resulted in a temporal divergence in OSN odorant responses (measured by the fluorescence of synaptopHluorin expressed in OSN axons) to a novel pair of similar odorants (Kass et al., 2016). Furthermore, fear conditioning has been demonstrated to result in the potentiation of the response to the paired odorant (Kass et al., 2013). Additionally, after odor-reward association learning, OSN responses to the trained odorants, which were monitored by intrinsic signal imaging, were shown to be potentiated (Abraham et al., 2014). We did not observe these changes in OSN activity. The source of this potential discrepancy is unclear and may be due to differences in behavioral contexts (including task difficulty and trial parameters), measurement methods (our GCaMP6s imaging vs synaptopHluorin signals or intrinsic signal imaging), choice of odorants used, etc. Nevertheless, in this and previous studies (Chu et al., 2016), we have compared the changes in OSN activity and mitral cell activity during the same behavioral task with the same odorants, providing a strong case for plasticity downstream of OSN inputs. We also note that our results do not exclude the potential contribution of changes in OSN activity that are not detected by our current method, such as changes in millisecond-level spike synchrony across OSNs, although it seems unlikely that such changes account for the entirety of the enhanced pattern separation observed in mitral cells.

There are many possible sites downstream of OSN input which could result in the changes in mitral cell activity during perceptual learning. For example, mitral cell activity is shaped by local inhibitory neurons, and plasticity in inhibitory connections could contribute to the enhanced pattern separation of mitral cell responses. Interestingly, thousands of newly generated inhibitory interneurons are integrated into the olfactory circuitry daily through adult neurogenesis, which provides an additional layer of inhibitory plasticity in the olfactory bulb (Alonso et al., 2006). Furthermore, the olfactory bulb receives ample feedback projections from higher brain centers. Cortical glutamatergic feedback, which mainly target inhibitory interneurons in the olfactory bulb, indirectly modulates mitral cell activity (Balu et al., 2007; Boyd et al., 2012; Markopoulos et al., 2012; Otazu et al., 2015; Mazo et al., 2016). Additionally, feedback from various neuromodulatory areas can shape mitral cell odor responses and have been demonstrated to play an important role in olfactory tasks such as odor discrimination and odor detection (Linster et al., 2001; Doucette and Restrepo, 2008; Chaudhury et al., 2009; Escanilla et al., 2010; Ma and Luo, 2012; Nunez-Parra et al., 2013; Kapoor et al., 2016). Future studies are needed to determine the specific loci and nature of changes responsible for the observed changes in mitral cell activity.

## Synthesis

Reviewing Editor: Zoltan Nusser, Institute of Experimental Medicine Decisions are customarily a result of the Reviewing Editor and the peer reviewers coming together and discussing their recommendations until a consensus is reached. When revisions are invited, a fact-based synthesis statement explaining their decision and outlining what is needed to prepare a revision will be listed below. The following reviewer(s) agreed to reveal their identity: Venkatesh Murthy, Hokto Kazama.

The authors have recently reported that the odor responses of mitral cells in the mouse olfactory bulb sparsened as the mouse learned to discriminate similar odors, such that the population level representations of two discriminated odors became more separable over days. In this study, the authors provide compelling evidence that this is not accompanied by plastic changes of the presynaptic olfactory sensory neurons (OSNs). Although, this is a relatively modest step forward, but it is an important one because several previous reports have shown that the activity of OSNs does change over time under certain circumstances. The conclusion here is much stronger as the activity of OSNs and mitral cells was collected from mice that underwent the same training protocol and the data were collected under exactly the same experimental setup.

In agreement with the reviewers, I feel that this manscript requires only minor changes before publication. The following minor points should be addressed:

1. Their description of how they label glomeruli as “divergent” or “responsive” is somewhat confusing (lines 151 onwards). Why is criterion 2 needed for “responsive” classification?

What is the significance of using 0.2 as threshold?

2. This is more of suggestion. Since they never compare across different subjects, why did they restrict the number of glomeruli in their decoder vector to 14. They could use all of the responsive glomeruli for calculating this vector.

3. they use different scales for each and every glomerulus in figure 2C - I think it would be more easy to compare if they are all in the same scale.

4. p.4 and p.14. In the Introduction (p.4), the authors describe that changes in mitral cell responses (Chu et al., 2016) can be either due to plasticity in OSNs or the olfactory bulb circuits. The possibility of plasticity in the cortical feedback should be mentioned as well just like in the Discussion (p.14). Conversely, Discussion should not be limited to cortical feedback to be consistent.

5. Figure 2C. Because the aim here is to demonstrate the stability of OSN responses over time, it is clearer if traces for day 1 and 7 are shown together for each glomerulus as in Fig. 3B and C.

6. l.252. “..., mitral cell responses showed an enhanced separation of the representations of the similar odorants during the discrimination task.” The text is a bit awkward.

7. l.306. “...that are not be detected...”. “be” should be removed.

8. Several references lack the journal name (e.g. l.441, l.460) and the style is not consistent.

## Author Response

Synthesis of Reviews:

Synthesis Statement for Author (Required):

The authors have recently reported that the odor responses of mitral cells in the mouse olfactory bulb sparsened as the mouse learned to discriminate similar odors, such that the population level representations of two discriminated odors became more separable over days. In this study, the authors provide compelling evidence that this is not accompanied by plastic changes of the presynaptic olfactory sensory neurons (OSNs). Although, this is a relatively modest step forward, but it is an important one because several previous reports have shown that the activity of OSNs does change over time under certain circumstances. The conclusion here is much stronger as the activity of OSNs and mitral cells was collected from mice that underwent the same training protocol and the data were collected under exactly the same experimental setup.

In agreement with the reviewers, I feel that this manuscript requires only minor changes before publication. The following minor points should be addressed:

We thank the reviewers and editor for the encouraging comments.

1. Their description of how they label glomeruli as “divergent” or “responsive” is somewhat confusing (lines 151 onwards). Why is criterion 2 needed for “responsive” classification?

What is the significance of using 0.2 as threshold?

We followed the methods we developed in Chu et al. Neuron 2016. Criterion 2 ensures that small artifacts do not erroneously generate responsive glomeruli.

2. This is more of suggestion. Since they never compare across different subjects, why did they restrict the number of glomeruli in their decoder vector to 14. They could use all of the responsive glomeruli for calculating this vector.

Here we also followed the methods we developed in Chu et al. Neuron 2016. 14 is the smallest number of responsive glomeruli across all animals. In general, the decoder accuracy is higher with a larger number of cells/glomeruli. Therefore the absolute value of decoder accuracy is simpler to interpret when the number of glomeruli is clearly defined and kept constant.

3. they use different scales for each and every glomerulus in figure 2C - I think it would be more easy to compare if they are all in the same scale.

We appreciate this suggestion and acknowledge that there are pros and cons for both methods of plotting responses. We tend to use different scales for different cells/glomeruli when the variability of response sizes is large. In this example, glom 1 and glom 4 are ~7-fold different in their responses and therefore difficult to show in the same scale.

4. p.4 and p.14. In the Introduction (p.4), the authors describe that changes in mitral cell responses (Chu et al., 2016) can be either due to plasticity in OSNs or the olfactory bulb circuits. The possibility of plasticity in the cortical feedback should be mentioned as well just like in the Discussion (p.14). Conversely, Discussion should not be limited to cortical feedback to be consistent.

We agree with this comment and have modified the text accordingly. In the introduction we now simply state 'downstream of OSN inputs' to avoid the distinction between local plasticity and feedback. In the discussion, we now spell out both possibilities.

5. Figure 2C. Because the aim here is to demonstrate the stability of OSN responses over time, it is clearer if traces for day 1 and 7 are shown together for each glomerulus as in Fig. 3B and C.

For this figure, we have tried both ways of plotting and, while we appreciate this comment made by the reviewers, we would like to keep it as is. When we plot this while grouping odors rather than days, it is difficult to appreciate for example that glom 9 is a divergent glomerulus.

6. l.252. “..., mitral cell responses showed an enhanced separation of the representations of the similar odorants during the discrimination task.” The text is a bit awkward.

We thank the reviewers for this comment and have modified this sentence.

7. l.306. “...that are not be detected...”. “be” should be removed.

We thank the reviewers for catching this error, which has been corrected.

8. Several references lack the journal name (e.g. l.441, l.460) and the style is not consistent.

We apologize for the oversight. The references have been reformatted and the mistakes have been corrected.
